# Long-chain *n*-alkane biodegradation coupling to methane production in an enriched culture from production water of a high-temperature oil reservoir

**DOI:** 10.1186/s13568-020-00998-5

**Published:** 2020-04-07

**Authors:** Jing Chen, Yi-Fan Liu, Lei Zhou, Muhammad Irfan, Zhao-Wei Hou, Wei Li, Serge Maurice Mbadinga, Jin-Feng Liu, Shi-Zhong Yang, Xiao-Lin Wu, Ji-Dong Gu, Bo-Zhong Mu

**Affiliations:** 1 State Key Laboratory of Bioreactor Engineering and School of Chemistry and Molecular Engineering, East China University of Science and Technology, 130 Meilong Road, Shanghai, 200237 People’s Republic of China; 2 Research Institute of Daqing Oilfield Company Limited, PetroChina, Daqing, Heilongjiang 163712 People’s Republic of China; 3 School of Biological Sciences, The University of Hong Kong, Pokfulam Road, Hong Kong Special Administrative Region, People’s Republic of China; 4 Department of Chemical, Polymer and Composite Materials Engineering, University of Engineering and Technology, KSK Campus, Lahore, 54890 Pakistan; 5 Engineering Research Center of Microbial Enhanced Oil Recovery, East China University of Science and Technology, 130 Meilong Road, Shanghai, 200237 People’s Republic of China

**Keywords:** Anaerobic biodegradation, Long-chain *n*-alkanes, High-temperature oil/petroleum reservoir, Methanogenesis, Metabolic pathway

## Abstract

Paraffinic *n*-alkanes (C22–C30), crucial portions of residual oil, are generally considered to be difficult to be biodegraded owing to their general solidity at ambient temperatures and low water solubility, rendering relatively little known about metabolic processes in different methanogenic hydrocarbon-contaminated environments. Here, we established a methanogenic C22–C30 *n*-alkane-degrading enrichment culture derived from a high-temperature oil reservoir production water. During two-year incubation (736 days), unexpectedly significant methane production was observed. The measured maximum methane yield rate (164.40 μmol L^−1^ d^−1^) occurred during the incubation period from day 351 to 513. The nearly complete consumption (> 97%) of paraffinic *n*-alkanes and the detection of dicarboxylic acids in *n*-alkane-amended cultures indicated the biotransformation of paraffin to methane under anoxic condition. 16S rRNA gene analysis suggested that the dominant methanogen in *n*-alkane-degrading cultures shifted from *Methanothermobacter* on day 322 to *Thermoplasmatales* on day 736. Bacterial community analysis based on high-throughput sequencing revealed that members of *Proteobacteria* and *Firmicutes* exhibiting predominant in control cultures, while microorganisms affiliated with *Actinobacteria* turned into the most dominant phylum in *n*-alkane-dependent cultures. Additionally, the relative abundance of *mcrA* gene based on genomic DNA significantly increased over the incubation time, suggesting an important role of methanogens in these consortia. This work extends our understanding of methanogenic paraffinic *n*-alkanes conversion and has biotechnological implications for microbial enhanced recovery of residual hydrocarbons and effective bioremediation of hydrocarbon-containing biospheres.

## Key points

- Biotransformation of paraffinic *n*-alkanes taking place under methanogenic conditions inoculated with high-temperature reservoir water.
- C22-C30 *n*-alkanes biodegradation coupling to methane generation.
- Almost all added substrates were consumed.
- *Actinobacteria* becoming predominant in alkane-degrading cultures.

## Introduction

Linear alkanes, major and critical constituents of natural gas and crude oil, can be metabolized by microbial consortia in extremely anoxic conditions, as extensively demonstrated in various environments (Boll et al. 2018; Gieg 2018; Rabus et al. 2016). Consequently, in the presence of microbial communities, *n*-alkanes of different chain lengths, considered as environmental contaminants, can be extracted from the subsurface environments for recovery (Siegert et al. 2013). However, paraffinic *n*-alkanes, one of the components of linear alkanes, is generally solid at ambient temperatures, resulting in their difficulties in extraction, transportation and handling which may be tackled by the mechanical or physical means but usually comparatively costly (Azevedo and Teixeira 2003; Burger et al. 1981; Sanjay et al. 1995; Wentzel et al. 2007). Therefore, potential biodegradation from waxy *n*-alkanes to methane can be an environmentally friendly and effective biotechnology strategy for further energy recovery and utilization tracked from hydrocarbon-contaminated environments (Head et al. 2014).

The inert *n*-alkanes have been proposed to be initially activated through several alternative enzymatic reactions without oxygen as the reductant (Boll et al. 2018; Mbadinga et al. 2011). Among them, fumarate addition pathway has been proposed to be a predominant activation mechanism used by alkane-degrading bacteria studied so far and degradation is the addition of alkanes across the double bond of fumarate with the formation of alkylsuccinates catalyzed by glycyl radical enzyme (Callaghan et al. 2009, 2008; Cravo-Laureau et al. 2005; Grundmann et al. 2008; Ji et al. 2019; Rabus et al. 2001).

It is not surprising that methanogenic alkane biodegradation requires cooperative interactions of microorganisms including alkane-degrading bacteria, syntrophic bacteria and methanogenic archaea, which has been well reported (Dolfing et al. 2008). To date, although anaerobic microorganisms capable of oxidizing *n*-alkanes have been isolated or highly enriched from biospheres, information on methanogenic long-chain alkanes degradation remains poorly understood (Boll et al. 2018; Wilkes and Rabus 2018). Actually, (Caldwell et al. 1998) documented anaerobic biodegradation of long-chain C15–C34 *n*-alkanes from a weathered crude oil within 201-day-incubation inoculated with chronically hydrocarbon-contaminated marine sediments under sulfate-reducing conditions. Similar observation proposed biotransformation of C13–C34 *n*-alkanes in amendments with weathered oil under both sulfate-reducing and methanogenic conditions by microorganisms derived from an anoxic natural gas condensate-contaminated aquifer, revealing that the availability of exogenous electron acceptor is not a constraint on biodegradation of long-chain *n*-alkanes (Townsend et al. 2003). The hyperthermophilic sulfate-reducing archaeon, *Archaeoglobus fulgidus*, was demonstrated to oxidize C10–C21 *n*-alkanes through addition to fumarate (Khelifi et al. 2014).

With respect to solid *n*-alkane (> C17) recent studies which used paraffin (*n*-octacosane) as the sole carbon and energy sources under methanogenic condition enriched from hydrocarbon-contaminated aquifer sediments of low temperature, has proved paraffin degradation coupled with methane generation. Metagenomic analysis proposed that the paraffin molecular was most likely activated via fumarate addition pathway by syntrophic members of *Smithella* (Oberding and Gieg 2018; Wawrik et al. 2016). Furthermore, it was shown that putative alkylsuccinate synthase (*assA*) gene was found in paraffin-degrading enrichment cultures derived from hydrocarbon-impacted aquifer sediments, indicative of anaerobic long-chain *n*-alkane metabolism (Callaghan et al. 2010). Despite the fact that high molecular weight alkanes are solid at room temperature, they actually tend to be melted in the oil phase under high-temperature condition (Etoumi 2007; Sanjay et al. 1995). Thus, long-chain paraffin is theoretically expected to be metabolized by hydrocarbon-degrading consortia and subsequently converted into methane by syntrophic microorganisms and methanogenic archaea in deep subsurface of high temperature (Jones et al. 2008; Oberding and Gieg 2018). Nevertheless, knowledge regarding the anaerobic biodegradation of long-chain paraffin and possible microbial populations in thermophilic cultures under methanogenic conditions remain unclear (Callaghan 2013; Wentzel et al. 2007).

In this work, methanogenic long-chain (C22–C30) *n*-alkane-degrading enrichment cultures were established using production water of a high-temperature oil reservoir as the inoculum. Measurement of biogenic methane accumulation during more than 2 years of incubation in conjunction with characterization and quantification of residual substrates was performed to assess the potential biodegradability of long-chain *n*-alkanes. Metabolites and functional genes were also identified through chemical and molecular biological approaches. Other than that, composition and diversity of microbial populations based on Illumina Miseq Sequencing were investigated, for the purpose of exploring the substances conversion and metabolic activities in paraffinic *n*-alkane-dependent consortia.

## Experimental sections

### Establishment of methanogenic enrichment culture

Production water (named “BG”) sampled from a high-temperature oil reservoir, from Zhan 3 block of Shengli oilfield in Shandong, China, was used as the inoculum for methanogenic enrichment cultures. Anaerobic experimental incubations were set up in 120 mL of clean and sterile serum bottles, containing 10 mL of autoclaved basal medium and 40 mL of production fluids. The basal medium contained: (g/l): NaCl, 2.0; KCl, 0.5; MgCl_2_·6H_2_O, 0.4; NH_4_Cl, 0.25; CaCl_2_·2H_2_O, 0.10; KH_2_PO_4_, 0.20; and resazurin, 0.001. And the basal medium was supplemented with 1.0 mL trace elements and 1.0 mL vitamins stock solution, which was described previously (Xu et al. 2019).

A mixture of long-chain *n*-alkanes (with the purity of 98–99% GC), purchased from TCI, including docosane (C_22_H_46_), tetracosane (C_24_H_50_), hexacosane (C_26_H_54_), octacosane (C_28_H_58_) and triacontane (C_30_H_62_), was added (40 mg of each compound) as the sole carbon sources, named “E”. Control without additional alkanes (“C”) was prepared to be able to eliminate the contribution of indigenous substrates. Samples at day 322 were named “1”, and samples at day 736 were named “2”. So, “E1” and “E2” represented *n*-alkane-amended cultures at day 322 and 736; “C1” and “C2” represented substrate-unamended samples at day 322 and 736. Additionally, heat-killed control sterilized for three times were also cultured and amended with a mixture of five alkanes. All experimental incubations in serum bottles flushed with 99.99% N_2_ were sealed by butyl rubber stopper and aluminum seal to ensure the strictly anaerobic conditions. Each enrichment culture was in quadruplicate and incubated at 55 °C in the dark.

### Chemical analysis

The measurement of biogas at intervals in cultures was conducted by a 500-μL gas-tight syringe to withdraw 200 μL of headspace using gas chromatography (GC 9890B), as described previously (Chen et al. 2019).

Quantification of volatile fatty acids (VFAs) was analyzed by ion chromatograph (ICS-1100, USA). For determination of long-chain fatty acids (LCFAs), culture aliquot of E and C were extracted at the incubations of days 322 and 736, the extracted organic solvent soluble fractions were pre-treated and injected onto the Gas Chromatography-Mass Spectrometer (GC–MS), as previously reported (Liang et al. 2016).

Residual *n*-alkanes were quantified by additional cetyl chloride and extracted by *n*-hexane, according to the previous description (Wang et al. 2011). The stoichiometric equations (Symons and Buswell 1933) of *n*-C22, *n*-C24, *n*-C26, *n*-C28 and *n*-C30 were used to calculate the maximum theoretical methane yields during 736 days of incubation.

1 $$n - {\text{Docosane }}\left( {n - {\text{C22}}} \right){\text{ - - - - - C}}_{ 2 2} {\text{H}}_{ 4 6} + { 1}0. 5 {\text{ H}}_{ 2} {\text{O}} \to 5. 2 5 {\text{ CO}}_{ 2} + { 16}. 7 5 {\text{ CH}}_{ 4}$$

2 $$n - {\text{Tetracosane }}\left( {n - {\text{C24}}} \right){\text{ - - - - - C}}_{ 2 4} {\text{H}}_{ 50} + { 11}. 5 {\text{ H}}_{ 2} {\text{O}} \to 5. 7 5 {\text{ CO}}_{ 2} + { 18}. 2 5 {\text{ CH}}_{ 4}$$

3 $$n - {\text{Hexacosane }}\left( {n - {\text{C26}}} \right){\text{ - - - - - C}}_{ 2 6} {\text{H}}_{ 5 4} + { 12}. 5 {\text{ H}}_{ 2} {\text{O}} \to 6. 2 5 {\text{ CO}}_{ 2} + { 19}. 7 5 {\text{ CH}}_{ 4}$$

4 $$n - {\text{Octacosane }}\left( {n - {\text{C28}}} \right){\text{ - - - - - C}}_{ 2 8} {\text{H}}_{ 5 8} + { 13}. 5 {\text{ H}}_{ 2} {\text{O}} \to 6. 7 5 {\text{ CO}}_{ 2} + { 21}. 2 5 {\text{ CH}}_{ 4}$$

5 $$n - {\text{Triacontane }}\left( {n - {\text{C3}}0} \right){\text{ - - - - - C}}_{ 30} {\text{H}}_{ 6 2} + { 14}. 5 {\text{ H}}_{ 2} {\text{O}} \to 7. 2 5 {\text{ CO}}_{ 2} + { 22}. 7 5 {\text{ CH}}_{ 4}$$

### Modified Gompertz model

The methane production was evaluated by the modified Gompertz model (Eq. 6) to indicate the growth of microorganisms (Gu 2016). Assuming that the methane production rate is proportional to microbial activity in enrichment culture, the modified Gompertz model can be used to assess the lag phase, maximum methane yield and methane production rate (Xu et al. 2019).

6 $$Y = {\rm A} \times exp\left\{ { - { \exp }\left( {K \times \left( {\lambda - t} \right) + 1} \right)} \right\}$$

7 $$K = \frac{V_{max} \times e}{A}$$

where *Y* = accumulative methane production (μmol), *A* = methane yield potential (μmol), *V*_*max*_= the maximum methane production rate (μmol/d), *e* = natural constant (2.7183), *λ* = lag phase time (days), *t* = the incubation time (days). *A*, *K* and *λ* could be estimated based on a statistical model of SGompertz using ORIGINPRO 8.0 software (OriginLab, USA). And *V*_*max*_ can be estimated by the Eq. (7).

### Characterization of microbial communities

Total genomic DNA samples were extracted from 5 mL enrichment cultures at 322 and 736 days in serum bottles following the instruction of AxyPrep™ Bacterial Genomic DNA Miniprep Kit protocol (Axygen Bicosciences, Inc., CA, USA), and stored at − 20 °C prior to further analysis.

Polymerase chain reaction (PCR) amplification of bacterial 16S rRNA genes V4–V5 regions was conducted using the primer set 515F (5′-GTGCCAGCMGCCGCGG-3′) and 907R (5′-CCGTCAATTCMTTTRAGTTT-3′), and 344F (5′-ACGGGGYGCAGCAGGCGCGA-3′) and 915R (5′-GTGCTCCCCCGCCAATTCCT-3′) for archaeal 16S rRNA genes. The PCR system and program were previously described (Li et al. 2017). All valid sequences obtained by Illumina Miseq sequencing were clustered into operational taxonomic units (OTUs) using the Silva database. The data of bacteria and archaea were deposited into the NCBI SRA database under the accession number PRJNA545407.

### Detection and phylogenetic analysis of *mcrA* genes

The methyl coenzyme-M reductase subunit alpha (*mcrA*) genes were amplified by the primer set mlas-mod-F and mcrA-rev-R (Angel et al. 2011). The PCR condition was conducted as follows: initial denaturation at 96 °C for 3 min, followed by 32 cycles of 96 °C for 30 s, 54 °C for 30 s and 72 °C for 45 s, and a final elongation for 10 min at 72 °C. After purifying, cloning and sequencing, valid sequences with more than 96% similarity were classified into operational taxonomic units (OTUs) through CD-HIT Suite and were translated through EMBOSS Transeq Bioinformatics tools. Sequences closely related to the representative OTUs were selected using BLAST in the GenBank database. Phylogenetic trees were constructed using MEGA7.0 software with neighbor-joining method and 1000 bootstrap replicates (Kumar et al. 2016). All raw *mcrA* gene sequences were submitted to GenBank under the accession numbers MK990831 to MK991144.

### Quantitative PCR of *mcrA* genes

Quantitative PCR of *mcrA* genes were conducted with the primer set MLF (5′- GGTGGTGTMGGATTCACACARTAYGCWACAGC-3′) and MLR (5′- TTCATTGCRTAGTTWGGRTAGTT -3′) (Luton et al. 2002) by using the SYBR Green I Real-Time PCR with a CFX96 thermocycler (BioRad, USA). The quantitative PCR reaction was performed with a tenfold dilution series of plasmids containing target DNA sequences to establish the standard curve. The qPCR reaction with a volume of 10 μL contained TB Green Premix Ex Taq II (5 μL), Bovine Serum Albumin solution (0.1 μL), each PCR primer (0.2 μL), ddH_2_O (3.5 μL) and DNA template (1 μL). The conditions were as follows: a pre-denaturation at 95 °C for 3 min, followed by 40 cycles of denaturation at 95 °C for 20 s, annealing at 55 °C for 30 s, elongation at 72 °C for 60 s.

## Results

### Gas production in enrichment cultures

**Fig. 1:**
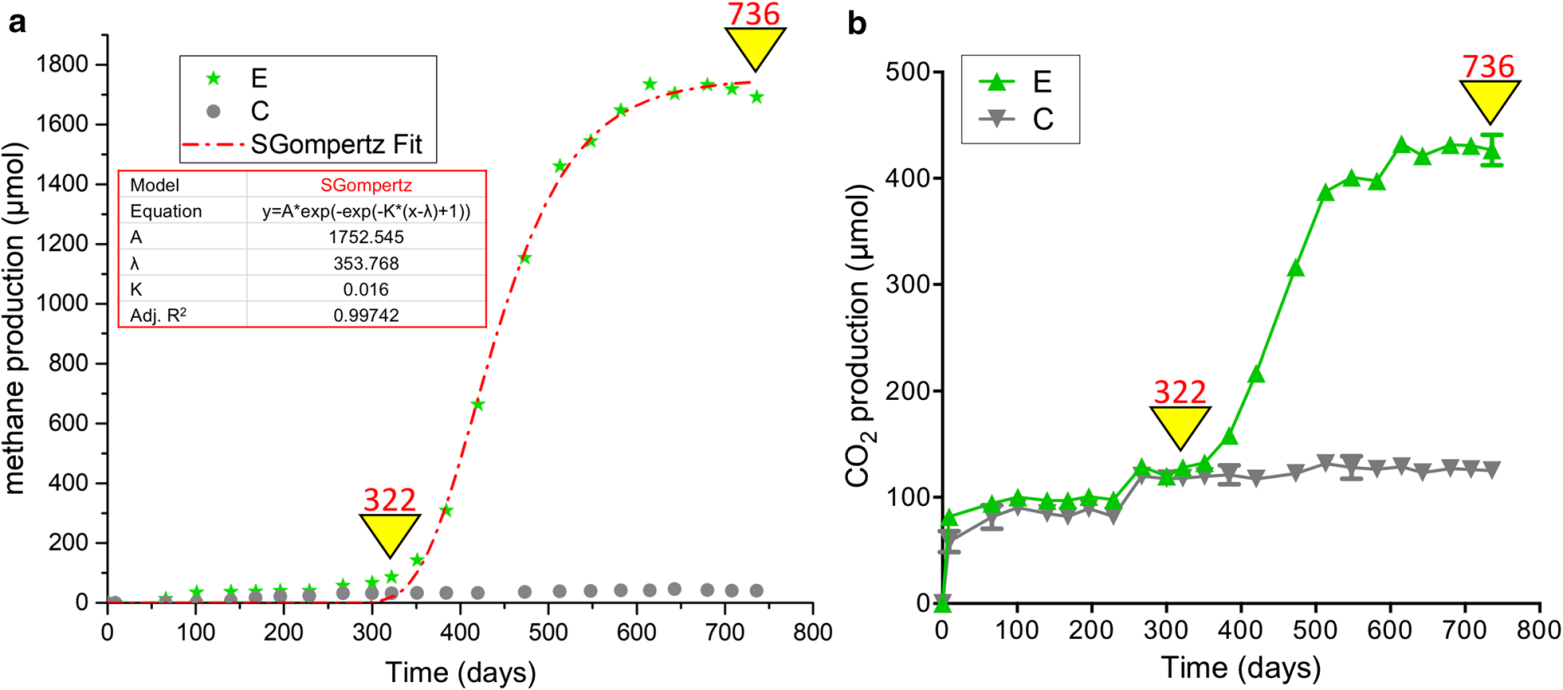
Accumulative production of CH_4_ (**a**) and CO_2_ (**b**) in enrichment cultures amended with long-chain *n*-alkanes (E) and control cultures without alkanes (C) inoculated with production water during 736-day incubation and the predicted curve of methane production (red line in **a**) with kinetic parameters using the modified Gompertz model

Over the 736 days of incubation, gas sampling in cultures E and C was periodically performed to monitor the accumulation of biogas, including methane, carbon dioxide and hydrogen. Accumulative methane formation and carbon dioxide was exhibited (Fig. 1), while hydrogen was under the detection limit in these two cultures.

As shown in Fig. 1a, enhanced methane production was observed in culture E amended with *n*-alkanes in comparison with that in culture C. After 322 days of incubation, 33 μmol of CH_4_ was detected in the substrate-free culture (C) and the methane generation then reached a plateau of approximately 43 μmol. By contrast, cultures amended with *n*-alkanes showed 72 μmol of methane at day 322, and then started to produce significant amount of CH_4_. A maximum methane production of 1754 μmol was observed in culture E at the end of incubation, suggesting that 1682 μmol of CH_4_ was generated at the second stage. CO_2_ started to accumulate at the beginning of the incubation in both cultures (Fig. 1b). Compared with the control sample without *n*-alkanes, an increasing amount of CO_2_ was observed in culture E after 322-day incubation, reaching from 128 μmol on day 322 to 427 μmol on day 736. Also, no production of methane and carbon dioxide were detected from the killed control culture (not shown in Fig. 1). The average production rate of methane was 0.12 and 4.06 μmol/d during the first and the second stage, respectively. Interestingly, it was obvious that during the incubation time from day 351 to day 513, the methane was quickly generated (from 142 to 1473 μmol). The measured maximum methane production rate (8.22 μmol/d) was observed at this period. It was estimated that the measured maximum methane yield rate was 164.40 μmol L^−1^ d^−1^ resulting from the 50-mL volume of the enrichment culture.

In addition, the predicted kinetic curve of methane production over the whole incubation time based on modified Gompertz modelling, to assess the predicted methane yield and methane production rate, was shown in Fig. 1a. It can be seen that the lag phase (*λ*) was 353.8 days and the methane yield potential (*A*) was 1752.54 μmol. According to the Eq. (7), the predicted maximum methane production rate was predicted to be 10.32 μmol/d (206.40 μmol L^−1^ d^−1^).

### Depletion of *n*-alkane and detection of intermediate metabolites

**Fig. 2:**
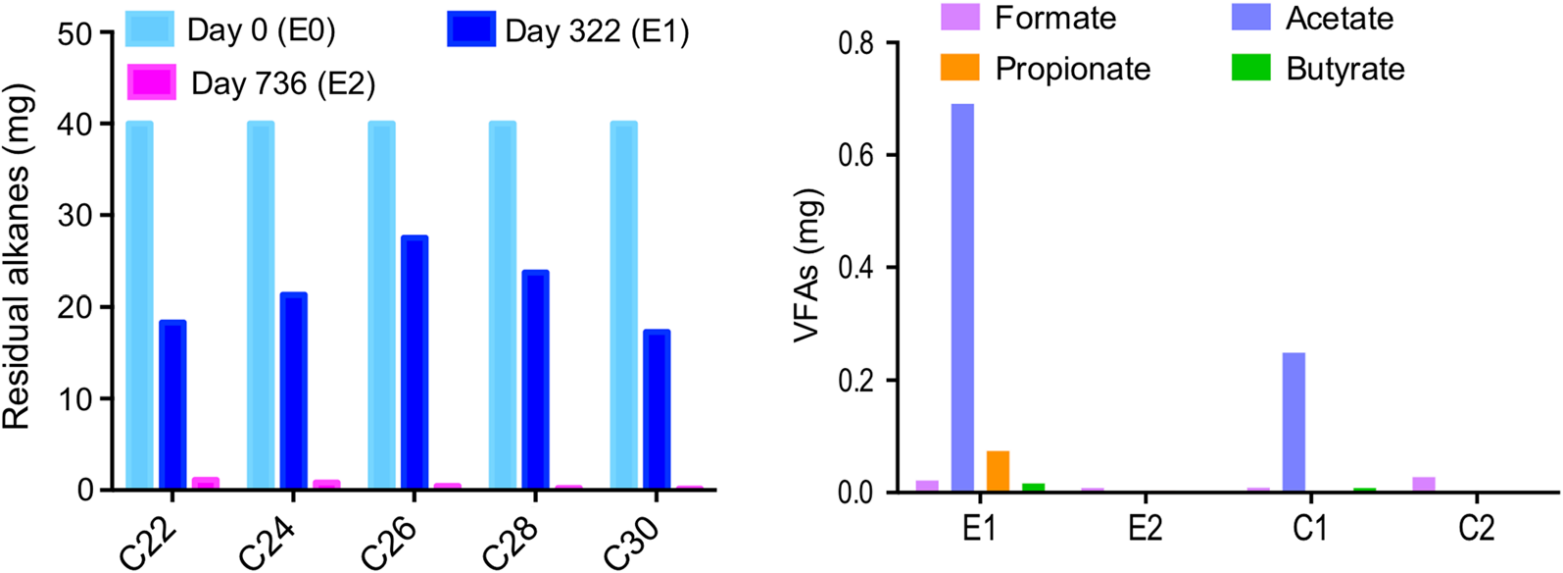
Residual alkanes and volatie fatty acids (VFAs) in enrichment cultures after anaerobic incubation

At the two stages of anaerobic incubation (day 322 and day 736), control and experimental samples were monitored for residual alkanes and metabolites. The residual alkanes and volatile fatty acids (VFAs) were shown in Fig. 2. Significant depletion of *n*-alkanes was observed in the culture E at different stages, especially at the second stage. Each added compound was consumed partly (31.3–56.8%) by 322 days and all added *n*-alkanes were consumed nearly completely (97.3–99.7%) at the end of the incubation. According to stoichiometric Eqs. (1–5), the consumption of *n*-alkanes during the incubation period were predicted to generate 10.09 mmol methane, which hence suggested the measured CH_4_ yield accumulated in headspace (1680.03 μmol) accounted for 16.7% of the theoretical value at 736 days (Additional file 1: Table S1).

No putative alkylsuccinates, which is the production of alkane addition to fumarate, were detected in any cultures, and this is probably due to their extremely slow microbial transformation and low steady state (Oberding and Gieg 2018). Neither did other putative bio-signature metabolites involved in other enzymatic activation mechanisms such as hydroxylation and carboxylation pathways. However, dicarboxylic acids (ranging from C4 to C12) that are probably involved in pathways downstream of fumarate addition can be found in *n*-alkane-amended samples except for C11 in E1 sample, and their mass spectra were shown in Additional file 1: Fig. S1.

### Diversity of microbial community composition

To study bacterial communities in the samples, over 35,000 valid sequences were acquired from Illumina Miseq sequencing for each sample (BG, E1, E2, C1 and C2). The relative abundance of quality sequences at the phylum level identified in these samples were used for bacterial community analysis shown as bubble plot (Additional file 1: Fig. S2). At the phylum level, the most abundant phylum in the inoculum sample and incubated samples were different. Among all bacteria in BG, sequences belonging to the phylum *Proteobacteria* were the most abundant group which represented nearly 90% of all valid sequences, and *Actinobacteria* and *Bacteroidetes* accounted for approximately 1.0% and 8.0%, respectively. Minor members in BG includes *Firmicutes*, *Acetothermia*, *Chloroflexi* and *Aminicenantes* (a total of less than 0.1%). It is evident that a decreasing relative abundance in *Proteobacteria* was detected in enrichment cultures compared with the BG. In culture C, the phylum *Firmicutes* contributed the most abundant taxa in culture C, constituting around 45.7% and 56.6% of all sequences in samples C1 and C2. Members of *Proteobacteria* showed the second highest relative abundance, comprising 39.0% and 36.4% at day 322 and 736, respectively. Interestingly, there was an obvious increase in the relative abundance of *Actinobacteria* in treatment E amended with *n*-alkanes during the incubation period. The relative abundance of sequences from this phylum increased from 20.2% of E1 to 55.8% of E2, which made *Actinobacteria* the dominant bacteria in culture E.

For archaeal communities, more than 30,000 high-quality sequences were obtained for each sample after 322 and 736 days of anaerobic incubation and were classified at the genus level. As shown in Additional file 1: Fig. S3, the dominant member in the inoculum sample and substrate-unamended control culture was *Methanothermobacter*, comprising 78.7% in BG, 98.5% in culture C1 and 90.8% in culture C2. While in culture E, the most abundant taxa shifted from *Methanothermobacter* at day 322 (49.0%) to sequences belonging to the order *Thermoplasmatales* at day 736 (71.0%).

### Phylogenetic analysis and quantification of *mcrA* genes

**Fig. 3:**
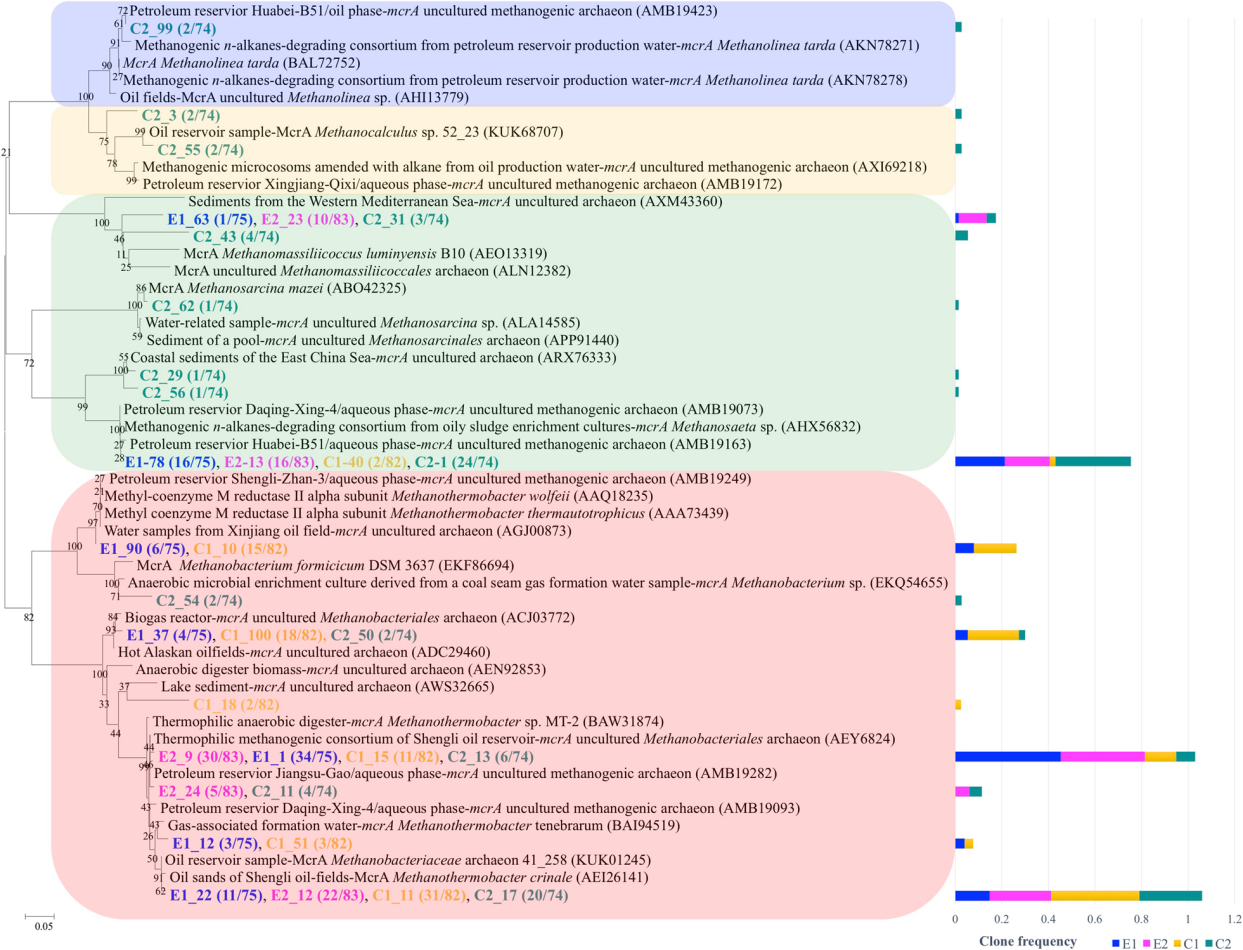
The phylogenetic tree of *mcrA* gene in cultures enriched from production water amended with long-chain *n*-alkanes during 736-day incubation

75, 83, 82 and 74 (in total of 314) *mcrA* sequences were recovered from cultures E1, E2, C1 and C2, respectively. All *mcrA* gene clones were grouped into 17 OTUs for phylogenetic analysis. As can be seen from the phylogenetic tree (Fig. 3), 17 OTUs could be classified into four different orders including *Methanomicrobiales* (in purple background), *Methanomassiliicoccus* (in yellow background), *Methanosarcinales* (in green background) and *Methanoabcteriales* (in red background). It was shown that over time, the diversity of predominant *mcrA* gene group increased in *n*-alkane-amended culture, but sequences of the order *Methanobacteriales* was still the dominant group of *mcrA* gene in each sample. Clone sequences belonging to the order of *Methanosarcinales* in cultures E1 (21.3%) and E2 (19.3%) were all affiliated with *Methanosaeta*. Representative OTUs E1_63 and E2_23 accounted for 1.3% and 12.0% of respective total sequences, closely related to *mcrA* sequences of *Methanomassiliicoccus*, which was not detected in results of the archaeal 16S rRNA gene.

**Fig. 4:**
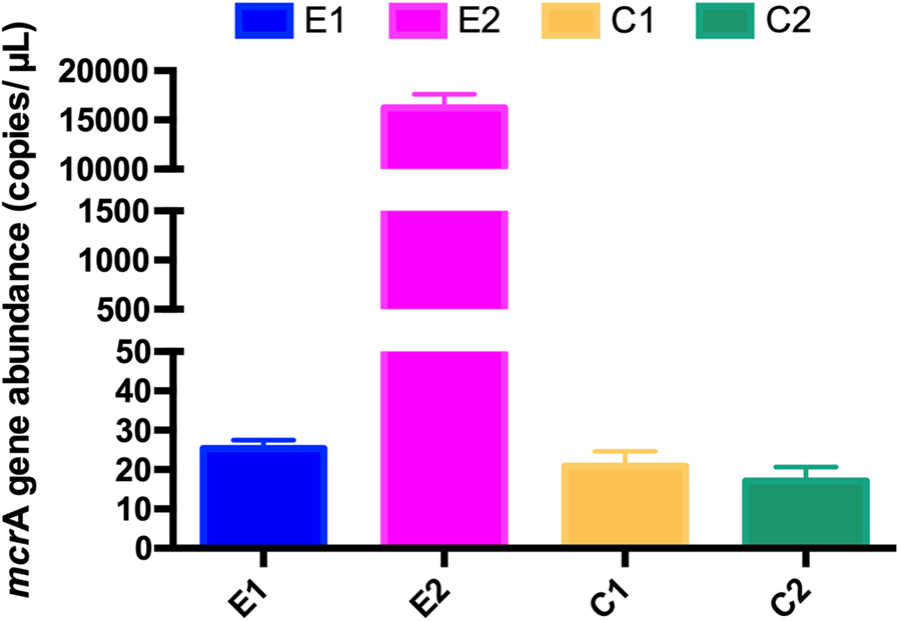
The *mcrA* gene abundance in enrichment cultures (column blue: E1; pink: E2; yellow: C1; green:C2) amended with long-chain *n*-alkanes

The qPCR analysis of *mcrA* gene indicated that gene abundance (gene copies per microliter of culture aliquot) of *mcrA* gene in E1, E2, C1 and C2 were 25.05, 1.63 × 10^4^, 20.67 and 16.92 (copies/μL), respectively (shown in Fig. 4). Obviously, *n*-alkane-degrading enrichment culture at the end of the incubation (the treatment E2) was detected with the most *mcrA* gene copies, which was consistent with the profile of methane production.

## Discussion

### Methanogenic biodegradation in C22–C30 *n*-alkane-dependent cultures

*n*-Alkanes have been reported to be converted to methane facilitated by microbial degradation under anoxic condition (Zengler et al. 1999). Having been studied for decades, the ubiquitous presence of methanogenic biotransformation of residual hydrocarbons promotes them to be important roles in the process of hydrocarbon contaminants conversion and recovery (Head et al. 2014). Despite increasing evidence that supports the potential of anaerobic microbial biodegradation of long-chain *n*-alkanes, the degradation of paraffinic *n*-alkanes is still considered challenging for microorganisms due to their insolubility at room temperature (Boll et al. 2018; Jimenez et al. 2016; Wilkes and Rabus 2018).

In the current study, a significant accumulation of methane can be observed in the long-chain *n*-alkane-dependent consortium derived from a high-temperature petroleum reservoir production water over a long term of approximately 2 years of anaerobic incubation. Combined with the almost complete consumption of added *n*-alkanes in experimental group (Fig. 2), microbial communities in E cultures were suggested to have the capability to degrade C22–C30 *n*-alkanes to methane, which is consistent with the research that paraffinic alkanes as the sole substrates could be converted to methane by a low-temperature methanogenic consortium enriched from hydrocarbon-contaminated aquifer sediments (Wawrik et al. 2016).

Furthermore, the detection of other metabolites including VFAs and dicarboxylic acids in *n*-alkane-amended cultures indicated that long-chain *n*-alkanes were probably metabolized to acids by anaerobic consortium and finally converted to methane and carbon dioxide. This proposed mechanism is similar to that which has been proposed for hexadecane degradation (Embree et al. 2014). Similar observation was reported in a methanogenic waxy hydrocarbon-degrading consortium from freshwater hydrocarbon-impacted aquifer sediments, where the consumption of *n*-octacosane (C28) was coupled with methane production and detection of several dicarboxylic acids (Oberding and Gieg 2018).

### Microbial populations acclimatize to long-chain *n*-alkanes over time

Methane was quickly formed and the culture rapidly reached the maximum methane production after initial active methanogenesis. Recent studies have demonstrated that the extent of microbial degradation of heavier *n*-alkanes is larger than that of lighter ones under anoxic condition (Cheng et al. 2014, 2019; Hasinger et al. 2012; Mishra et al. 2017). Added *n*-alkanes were consumed almost completely (97.3–99.7%) by the end of the cultivation, but theoretical methane production was relatively low (Additional file 1: Table S1). Hence, further transfer enrichments are expected to obtain the determination and quantification of metabolites to address the metabolic fate of paraffin.

As indicated by amplicon survey and qPCR analysis of *mcrA* functional gene, the relative abundance of *mcrA* gene in cultures amended with paraffinic *n*-alkanes increased significantly from day 322 to day 736. It is likely that hydrogentrophic methaogenesis may become the major methanogenic pathway, as suggested by phylogenetic analysis of *mcrA* genes (Fig. 3), which presented the highest abundance of *Methanothermobacter* in alkane-degrading cultures, especially in E2 (Khelifi et al. 2014; Mayumi et al. 2011).

In addition, a clear variation of microbial community structure was observed during the nearly two-year-incubation period, also indicating an acclimatization to the long-chain *n*-alkanes over the cultivation (Liang et al. 2016). The evident that higher frequency of the phylum *Actinobacteria* in long-chain *n*-alkane-dependent cultures after a long period of anaerobic incubation may suggest a potential role in the conversion of C22–C30 *n*-alkanes to methane (Additional file 1: Fig. S2). Although members of *Actinobacteria* were widely known for aerobic respiration (Barka et al. 2016; Kunapuli et al. 2007), they were observed in other anoxic hydrocarbon-degrading cultures (Mbadinga et al. 2012; Wang et al. 2012; Xu et al. 2019), and identified to be major phylogenetic groups in anaerobic bisphenol A degrading sediments (Yang et al. 2015) and were hypothesized to participate in methanogenic glucose degradation in anaerobic digester sludge (Ito et al. 2012). Besides, the phylum *Actinobacteria* was implicated to utilize secondary substrates or dead biomass as the primary syntrophs in an iron-reducing benzene-degrading enrichment culture with the help of stable isotope probing (Kunapuli et al. 2007). Accordingly, the absolute dominance of the phylum *Actinobacteria* probably suggested their involvement in the microbial metabolism from waxy *n*-alkanes to methane. Nevertheless, additional further study is required to explore the crucial players and active microorganisms in high-temperature long-chain alkane-degrading cultures.

### Putative paraffin degradation pathway in thermophilic methanogenic paraffin-dependent enrichments

Recent functional information and chemical and metagenomic analysis of methanogenic paraffin biodegradation indicated that *n*-octacosane can be initially activated to alkylsuccinates via the fumarate addition pathway by uncultured members of *Smithella* in *n*-alkane-degrading consortia, retrieved from low-temperature fuel-contaminated aquifer sediments (Callaghan et al. 2010; Wawrik et al. 2016). However, in this study, the predominant bacterial members in C22–C30 *n*-alkane-dependent cultures were affiliated to *Actinobacteria*, and members of *Actinobacteria* were not known for anaerobic alkane biodegradation. To further verify the lack of canonical *assA* genes in our samples, we utilized more than ten previously designed sets of different primers targeting canonical *assA* genes with a broad diversity (Abu Laban et al. 2015; Callaghan et al. 2010; Gittel et al. 2015; von Netzer et al. 2013). However, no amplicons could be retrieved here, implying that either there lack such genes in the consortia, or the *assA* genes here are too diverse to be targeted by the primers that we used (Cheng et al. 2019). Although no alkylsuccinate-like metabolites were acquired from the samples, dicarboxylic acids were detected only in long-chain *n*-alkane-amended cultures. According to previously postulated mechanism (Oberding and Gieg 2018), in which both ends of paraffin can be activated by the fumarate addition pathway. Future studies such as the design of new and universal primers as well as SIP techniques (Cheng et al. 2013; Jimenez et al. 2016; Kleindienst et al. 2014; Kunapuli et al. 2007) are required to unravel the exact activation mechanisms of paraffin.

On the other hand, other possible enzymatic activation pathways including hydroxylation, carboxylation, formation of alkyl-coenzyme-M and ‘intra hydroxylation’ oxidation should not be overlooked (Callaghan et al. 2009; Heider and Schühle 2013; Laso-Pérez et al. 2016; Rabus and Heider 1998; Zedelius et al. 2011). Similar observations were made in a methanogenic consortium enriched from Shengli oilfield amended with heavy oil (Cheng et al. 2019). Since there still remained uncertain with respect to the key active microorganisms and activation pathways in this work, more microbial and functional information are expected to be explored via omics-based sequencings and other chemical techniques (Gieg and Toth 2016; von Netzer et al. 2018). And also, there may exist novel pathway documented recently based on metagenomics analysis (Liu et al. 2019).

The current available information about biotransformation of paraffin in thermophilic anoxic environments is limited. In this work, we obtained a methanogenic long-chain (C22–C30) *n*-alkane-degrading enrichment culture inoculated from production water of a high-temperature oil reservoir. Significant production of methane and almost complete consumption of substrates in *n*-alkane-amended cultures indicated that substrates consumption was coupled to methane production. Additionally, high-throughput sequencing analysis suggested that members of *Actinobacteria* turned to be predominant in *n*-alkane-amended cultures after 736 days of incubation. However, active microorganisms and metabolic pathways require further evidence, probably by using metagenomics and metatranscriptomics sequencing and stable isotope probing techniques. These observations enriched our current knowledge of metabolic processes in anaerobic biodegradation of paraffinic alkanes under high-temperature conditions.

## Supplementary information

**Additional file 1: Table S1.** Predicted and measured methane (CH4) yield from enrichment cultures amended with *n*-alkane mixtures incubated after 322 and 736 days. **Fig. S1.** Mass spectral profiles of dicarboxylic acids identified in (A) E1 and (B) E2 cultures (Diethyl esterification derivatives). **Fig. S2.** Bubble plot of bacteria at the phylum level showing the relative abundance in enrichment cultures after incubations (BG: inoculum; E1 and E2: *n*-alkanes-amended cultures at day 322 and 736; C1 and C2: un-amended samples at day 322 and 736; Red: < 30%; Yellow: 30–80%; Green: ≥ 80%). **Fig. S3.** Bubble plot of archaea at the genus level showing the relative abundance in enrichment cultures after incubations (BG: inoculum; E1 and E2: *n*-alkanes-amended cultures at day 322 and 736; C1 and C2: un-amended samples at day 322 and 736; Red: < 30%; Yellow: 30–80%; Green: ≥ 80%).

## Data Availability

Raw data for microbial community sequencing and *mcrA* genes sequences are available in the GenBank archive at the National Center for Biotechnological Information (NCBI) as listed in the manuscript.

## Abbreviations

*assA*: Alkylsuccinate synthase gene
*mcrA*: Methyl coenzyme-M reductase gene
BG: Production water as an inoculum
E1: *n*-Alkane-amended cultures at day 322
E2: *n*-Alkane-amended cultures at day 736
C1: Substrate-unamended cultures at day 322
C2: Substrate-unamended cultures at day 736
