# Supplementary material for: Long-chain n-alkane biodegradation coupling to methane production in an enriched culture from production water of a high-temperature oil reservoir
Source: AMB Express. 2020 Apr 7;10:63. doi: 10.1186/s13568-020-00998-5 (PMC7138878; doi:10.1186/s13568-020-00998-5)
Supplement: Supplementary file 1 — Additional file 1: Table S1. Predicted and measured methane (CH4) yield from enrichment cultures amended with n-alkane mixtures incubated after 322 and 736 days. Fig. S1. Mass spectral profiles of dicarboxylic acids identified in (A) E1 and (B) E2 cultures (Diethyl esterification derivatives). Fig. S2. Bubble plot of bacteria at the phylum level showing the relative abundance in enrichment cultures after incubations (BG: inoculum; E1 and E2: n-alkanes-amended cultures at day 322 and 736; C1 and C2: un-amended samples at day 322 and 736; Red: < 30%; Yellow: 30–80%; Green: ≥ 80%). Fig. S3. Bubble plot of archaea at the genus level showing the relative abundance in enrichment cultures after incubations (BG: inoculum; E1 and E2: n-alkanes-amended cultures at day 322 and 736; C1 and C2: un-amended samples at day 322 and 736; Red: < 30%; Yellow: 30–80%; Green: ≥ 80%). [file 13568_2020_998_MOESM1_ESM.pdf]

Supplementary Materials

AMB express

Long-chain *n*-alkane biodegradation coupling to methane production in an enriched culture from production water of a high-temperature oil reservoir

Jing Chen[1, 5], Yi-Fan Liu[1, 5], Lei Zhou[1, 5], Muhammad Irfan[1, 4], Zhao-Wei Hou[2], Wei Li[2], Serge Maurice Mbadinga[1, 5], Jin-Feng Liu[1, 5], Shi-Zhong Yang[1, 5], Xiao-Lin Wu[2 *], Ji-Dong Gu[3], Bo-Zhong Mu[1, 5 *]

[1] *State Key Laboratory of Bioreactor Engineering and School of Chemistry and Molecular Engineering, East China University of Science and Technology, 130 Meilong Road, Shanghai 200237, P.R. China*

[2] *Research Institute of Daqing Oilfield Company Limited, PetroChina, Daqing, Heilongjiang 163712, P. R. China*

[3] *School of Biological Sciences, The University of Hong Kong, Pokfulam Road, Hong Kong Special Administrative Region, P.R. China*

[4] *Department of Chemical, Polymer and Composite Materials Engineering, University of Engineering and Technology, KSK Campus, Lahore 54890, Pakistan*

[5] *Engineering Research Center of Microbial Enhanced Oil Recovery, East China University of Science and Technology, 130 Meilong Road, Shanghai 200237, P.R. China*

[*]Corresponding Author:

Bo-Zhong Mu, E-mail: bzmu@ecust.edu.cn

Xiao-Lin Wu, E-mail: wuxldq@petrochina.com.cn

Table S1 Predicted and measured methane (CH_4) yield from enrichment cultures amended with *n*-alkane mixtures incubated after 322 and 736 days

Alkanes		Time (days)	Substrate Consumed (mg)	Substrate Consumed (μmol)	Percent of substrate depletion	Predicted CH_4 Yield (μmol) §	Measured CH_4 Yield (μmol) *	Theoretical production CH_4 (%)
***n*-alkane mixtures**	C_{22}	322	21.68	68.49	54.2%	CH_4 4924.755	CH_4 39.45	CH_4 0.80%
	C_{24}		18.70	55.22	46.8%			
	C_{26}		12.46	33.99	31.3%			
	C_{28}		16.28	41.24	40.7%			
	C_{30}		22.72	53.72	56.8%			
	C_{22}	736	38.91	122.89	97.3%	CH_4 10089.245	CH_4 1680.03	CH_4 16.7%
	C_{24}		39.22	115.82	98.0%			
	C_{26}		39.58	107.94	99.0%			
	C_{28}		39.80	100.82	99.5%			
	C_{30}		39.87	94.29	99.7%			

* Calculated from the difference of measured initial methane production, taking into account the methane production in control group (day 322: 32.98 μmol; day 736: 43.17 μmol).

§ Calculated using Equations 1-5 (main text) and the calculated mass of substrate consumed.

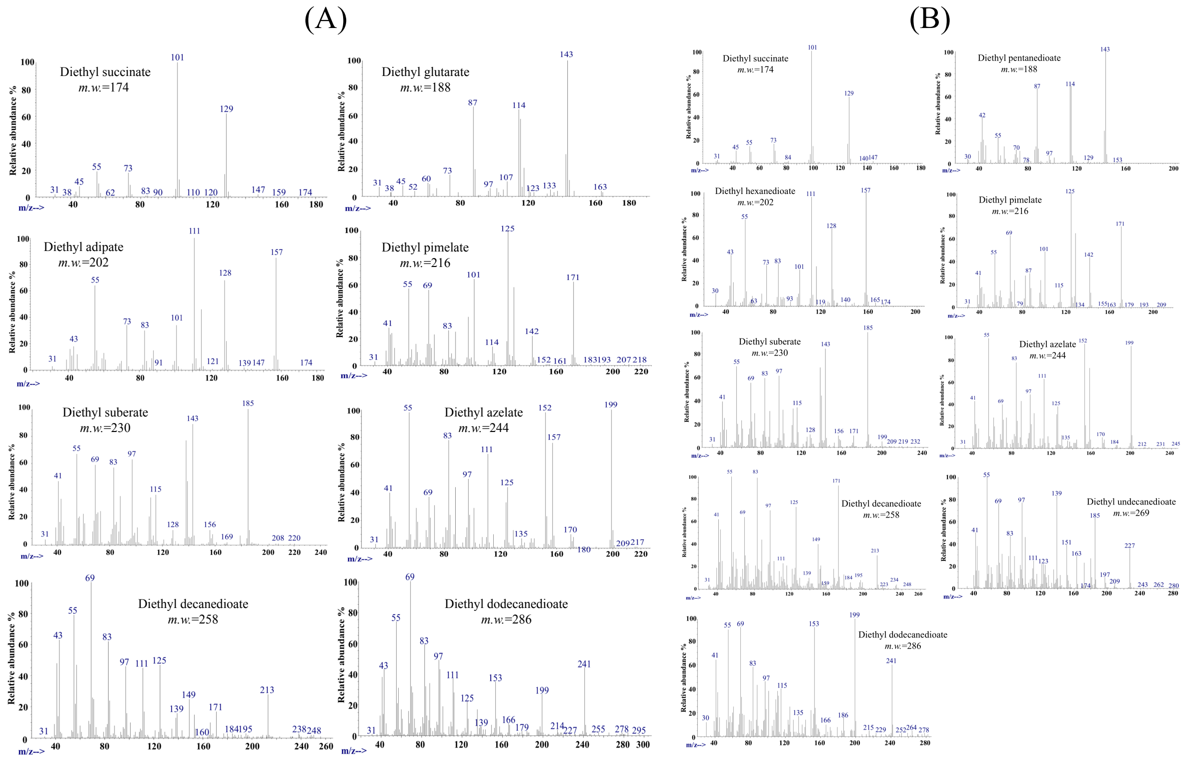

Fig. S1 Mass spectral profiles of dicarboxylic acids identified in (A) E1 and (B) E2 cultures (Diethyl esterification derivatives).

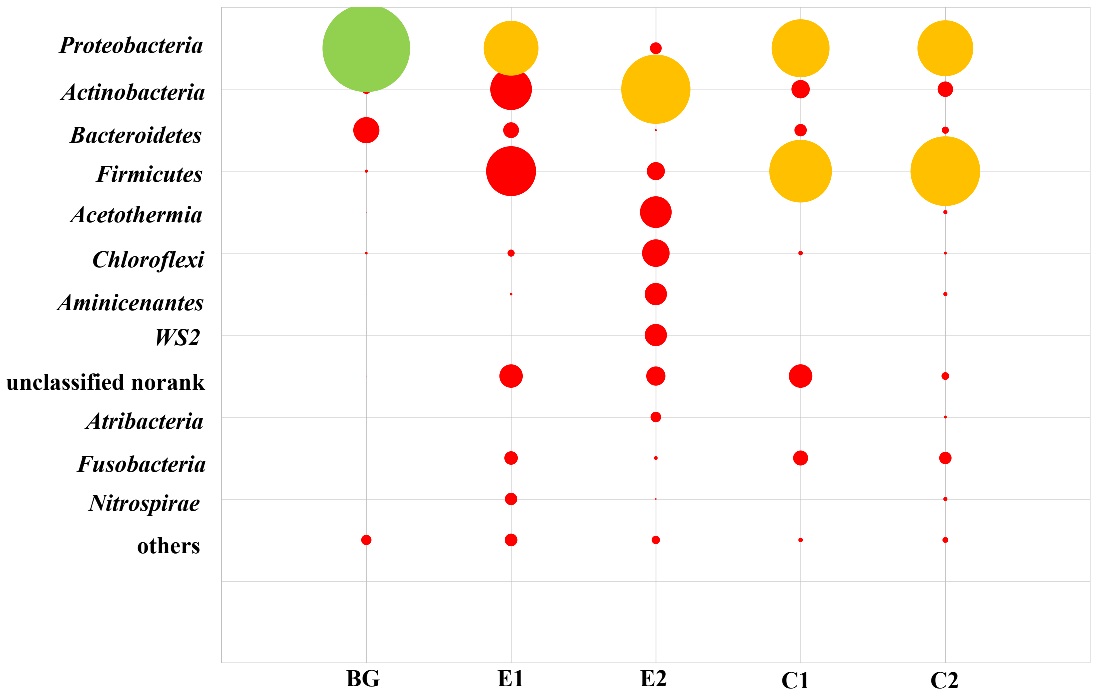

Fig. S2 Bubble plot of bacteria at the phylum level showing the relative abundance in enrichment cultures after incubations (BG: inoculum; E1 and E2: *n*-alkanes-amended cultures at day 322 and 736; C1 and C2: un-amended samples at day 322 and 736; Red: < 30%; Yellow: 30-80%; Green: >= 80%)

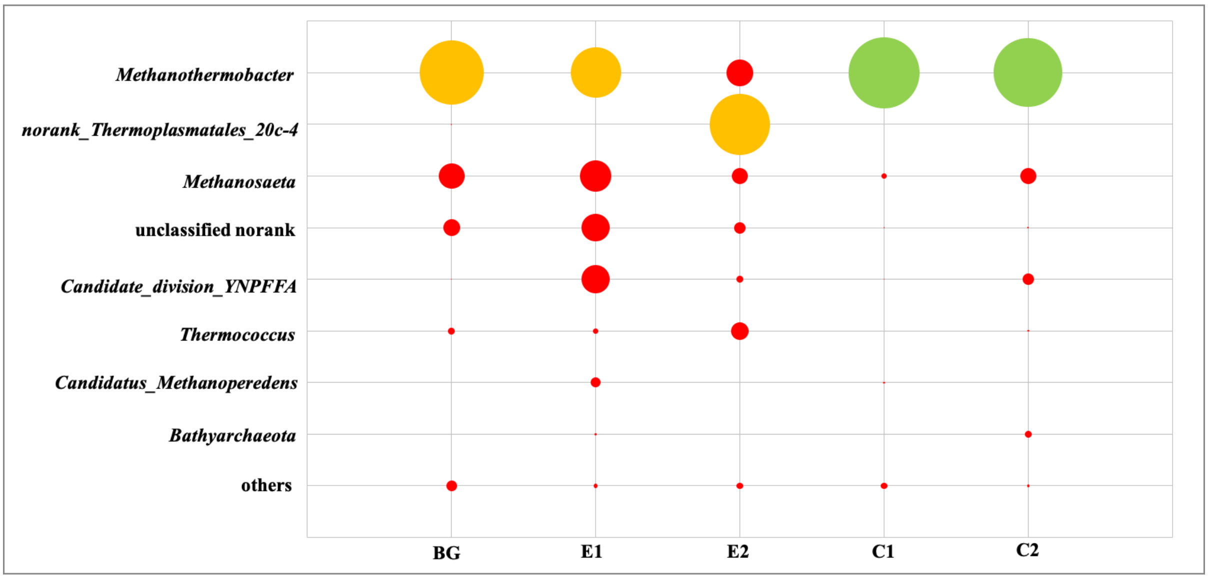

Fig. S3 Bubble plot of archaea at the genus level showing the relative abundance in enrichment cultures after incubations (BG: inoculum; E1 and E2: *n*-alkanes-amended cultures at day 322 and 736; C1 and C2: un-amended samples at day 322 and 736; Red: < 30%; Yellow: 30-80%; Green: >= 80%)
